# Dairy By-Products and Lactoferrin Exert Antioxidant and Antigenotoxic Activity on Intestinal and Hepatic Cells

**DOI:** 10.3390/foods12102073

**Published:** 2023-05-21

**Authors:** Inés Abad, Julien Vignard, Catherine Bouchenot, Dimitra Graikini, Laura Grasa, María Dolores Pérez, Gladys Mirey, Lourdes Sánchez

**Affiliations:** 1 Departamento de Producción Animal y Ciencia de los Alimentos, Facultad de Veterinaria, Universidad de Zaragoza, 50013 Zaragoza, Spain; 647018@unizar.es (I.A.); grekinid@gmail.com (D.G.); dperez@unizar.es (M.D.P.); 2 Instituto Agroalimentario de Aragón IA2 (UNIZAR-CITA), 50013 Zaragoza, Spain; lgralo@unizar.es; 3 Toxalim (Research Centre in Food Toxicology), Université de Toulouse, INRAE, ENVT, INP-Purpan, UPS, 31027 Toulouse, France; julien.vignard@inrae.fr (J.V.); catherine.bouchenot@univ-tlse3.fr (C.B.); 4 Departamento de Farmacología, Fisiología y Medicina Legal y Forense, Facultad de Veterinaria, Universidad de Zaragoza, 50013 Zaragoza, Spain

**Keywords:** buttermilk, whey, lactoferrin, oxidative stress, DNA damage, bioavailability, Caco-2 cells, HepG2 cells

## Abstract

The dairy industry generates a large volume of by-products containing bioactive compounds that may have added value. The aim of this study was to evaluate the antioxidant and antigenotoxic effects of milk-derived products, such as whey, buttermilk, and lactoferrin, in two human cell lines: Caco-2 as an intestinal barrier model and HepG2 as a hepatic cell line. First, the protective effect of dairy samples against the oxidative stress caused by menadione was analyzed. All these dairy fractions significantly reversed the oxidative stress, with the non-washed buttermilk fraction presenting the greatest antioxidant effect for Caco-2 cells and lactoferrin as the best antioxidant for HepG2 cells. At concentrations that did not impact cell viability, we found that the dairy sample with the highest antigenotoxic power against menadione, in both cell lines, was lactoferrin at the lowest concentration. Additionally, dairy by-products maintained their activity in a coculture of Caco-2 and HepG2, mimicking the intestinal-liver axis. This result suggests that the compounds responsible for the antioxidant activity could cross the Caco-2 barrier and reach HepG2 cells on the basal side, exerting their function on them. In conclusion, our results show that dairy by-products have antioxidant and antigenotoxic activities, which would allow revaluing their use in food specialties.

## 1. Introduction

The dairy industry generates a large volume of by-products whose current destination is mainly to obtain technological ingredients or become supplements for animal feeding. Considering that milk and its by-products, such as whey and buttermilk, contain proteins and other compounds with biological properties [1], their isolation would give them extra value and an additional source of income for the dairy industry.

Milk proteins are recognized as the main source of biologically active peptides, some displaying beneficial activities, such as antihypertensive, antidiabetic, antioxidant, immunomodulatory, and mineral binding properties [2]. In addition, milk anti-inflammatory properties can be attributed to the presence of phospholipids and short- or medium-chain saturated fatty acids [3].

Whey is obtained during cheese or casein manufacturing, and it has long been considered of little value. Nowadays, whey is used for animal feed in liquid form and as an ingredient in the food industry in dried form. The global production rate of whey in 2016 was estimated at 200 million tones (MT) per year, showing an annual increase of 3% [4]. Only in Europe were 57 MT of whey produced per year [5].

Whey contains about 20% of milk proteins, about 8% of fat, and 70% of lactose, making it an important source of nutrients [6]. The protein components of whey include β-lactoglobulin, bovine serum albumin, α-lactalbumin, immunoglobulins, lactoferrin, lactoperoxidase, other enzymes, and glycomacropeptide [7]. Whey proteins remain soluble after acid or enzymatic coagulation of milk, which distinguishes them from caseins that precipitate under these conditions [8]. Whey proteins have excellent functional properties, such as good solubility, viscosity, emulsifying, and gelling properties, and they are mainly responsible for the high nutritional and technological value of whey [9]. Thus, whey concentrates and isolates are widely used in the food industry [10]. A few years ago, there was an increased interest in some bioactive whey proteins and peptides, as they have health benefits and can be used as ingredients in functional foods [11,12]. The high content of proteins in whey is responsible for the high nutritional and technological value of this by-product [9]. Among them, lactoferrin (LF) is one of the main defensive proteins. LF is a cationic glycoprotein belonging to the transferrin family, with iron-binding capacity and numerous properties, such as antimicrobial activity and the ability to protect cells from oxidative stress [13]. LF may decrease the production of intracellular reactive oxygen species (ROS) or suppress the senescence of cells induced by hydrogen peroxide [14,15]. In 2012, bovine LF was allowed as a novel food ingredient under Regulation (EC) No 258/97 [16]. This regulation specified that bovine LF, as a protein that occurs naturally in cow’s milk, could be placed on the market as a novel food ingredient. In addition, this regulation establishes the limits for its use in different food categories, such as infant and follow-on formulas, beverages based on milk, etc. For infant formulas, the maximum use level proposed is 1 mg/mL, and for drink mixes based on milk, the indicated level is between 200 and 330 mg/100 g, depending on whether the product is in liquid or powder form. The broad properties of LF on human health have been reviewed recently [17,18].

Buttermilk (BM) is another dairy by-product released by butter manufacturing. Estimating the environmental impact through a life cycle assessment methodology, it has been observed that BM does not influence the impact caused by the butter manufacturing process in as high a proportion as whey does in the cheese production process [19]. For many years, BM has been undervalued, although some studies carried out in the last decade have shown its potential, especially due to the proteins and lipids present in the milk fat globule membrane (MFGM) [20]. MFGM is particularly rich in proteins and phospholipids and has great potential for functional and nutraceutical applications [9]. During churning in butter making, milk fat globules are disrupted, allowing the release of the fat enveloped by the MFGM and the phase inversion with the release of BM as the aqueous phase. BM is similar to whey in lactose content but contains a higher amount of protein [20]. Furthermore, BM contains a high amount of fat, with its phospholipid concentration being seven times higher than that of whole milk [21]. Considering the large volume of whey produced annually, BM derived from butter elaborated with whey fat has a huge potential market. In addition, BM has emulsifying properties due to the presence of caseins and considerable amounts of phospholipids, making it suitable for technological application in the food industry [22].

Seventy-one percent of all the whole milk available in European Union dairy is used for the manufacture of cheese and butter [5], which generates a large collateral production of whey and buttermilk. Numerous studies have clearly presented the valuable influence of whey, LF, and BM on human health and well-being. These fractions and some of their proteins have antimicrobial, antioxidant, antihypertensive, antidiabetic, or immunomodulatory properties [23,24,25,26]. The antioxidant, antimicrobial, and anticarcinogenic activities of bovine milk proteins, as well as the properties of their hydrolysates, have been reviewed recently [27]. With all this information gathered, it can be expected that the dairy fractions show some activity against reactive oxygen species and exert a protective role on DNA. The aim of this study was, therefore, to evaluate the antioxidant and antigenotoxic effects of milk-derived products, such as whey and BM, and compare them to LF, a milk protein with known antioxidant properties, in a hepatic and a colon carcinoma cell lines, simulating the conditions of the liver-intestine axis. In this study, we show evidence to consider milk fractions as potential antioxidants after oxidative stress caused by menadione. Consequently, this work intends to add value to the dairy by-products beyond their current applications as animal feed or complementary technological ingredients.

## 2. Materials and Methods

### 2.1. Chemicals and Preparation of Milk Samples

Calicheamicin (Pfizer, France) is a cytotoxic agent that causes double-strand DNA breaks, used as a positive control for viability assays and genotoxicity studies. Menadione (Sigma-Aldrich, St. Louis, MO, USA) was used to induce oxidative stress and N-acetylcystein (Sigma-Aldrich) as an antioxidant.

The dairy fractions used in this study for evaluating their bioactivity were obtained by different processes, as follows:

Raw bovine milk was supplied by the dairy company Villacorona (El Burgo de Ebro, Spain). The quality of milk was verified after reception by checking the pH, acidity, fat percentage, and alkaline phosphatase and lactoperoxidase activities, and it was processed as explained in a previous study [28] at the Food Science and Technology Pilot Plant of the University of Zaragoza, located in the Veterinary Faculty. The whey and BM obtained were lyophilized and kept at −20 °C for later use. After freeze-drying, the concentration of whey obtained was 0.068 g of dry matter per mL and that of BM was 0.029 g of dry matter/mL. In addition, by performing a bicinchoninic acid test, the amount of protein present in each of these samples was analyzed, obtaining 153.8 mg of protein per g of whey and 121.8 mg of protein per g of BM.

In the process of making BM, a step of cream washing was included to reduce the content of milk proteins, based on the method described by Le et al. [29]. Cream obtained from raw milk was washed twice with four volumes of phosphate-buffered saline (PBS), composed of 140 mM NaCl, 8 mM Na_2_HPO_4_, 1.5 mM KH_2_PO_4_, 2.7 mM KCl, pH 7.4, and 1 mM ethylenediaminetetraacetic acid (EDTA), centrifuging between each wash at 3000× *g* for 15 min at 4 °C. Washed cream was stirred to obtain butter grains, and washed buttermilk (WBM) was obtained, lyophilized, and kept at −20 °C.

Bovine LF was kindly donated by Tatua Nutritionals (Morrinsville, New Zealand). This LF was used in our previous assays, in which iron saturation, purity, and content of LPS were determined [30].

All samples were diluted in PBS to obtain a concentration of 40 mg/mL and filtered with 0.22 µm low-binding protein Millipore filters (Merck, Darmstad, Germany). The final concentrations of LF, whey, and BM used in the assays were 10, 5, 2, 1, and 0.5 mg/mL. All samples were tested in duplicate in each assay.

### 2.2. Cell Culture

The cell lines used in this study were HepG2, a human liver cancer cell line, and Caco-2, a human colon carcinoma cell line, both obtained from the American Type Culture Collection (ATCC, Manassas, VA, USA). HepG2 cells were maintained with Eagle’s Minimum Essential Medium (MEM) (Thermo Fisher Scientific, Rockford, IL, USA), supplemented with 10% (*v*/*v*) fetal bovine serum (FBS) (Thermo Fisher Scientific), 1% (*v*/*v*) antibiotics (penicillin 10,000 units/mL-streptomycin 10 mg/mL, Thermo Fisher Scientific), and 1% (*v*/*v*) 200 mM L-glutamine (Thermo Fisher Scientific). Caco-2 cells were maintained with Dulbecco’s Modified Eagle’s Medium (DMEM) with glutamine (Thermo Fisher Scientific) supplemented with 20% (*v*/*v*) FBS and 1% (*v*/*v*) antibiotics. Caco-2 cells were differentiated for 17 days as previously described [31].

The cells were cultured at 37 °C in a 5% CO_2_ humidified atmosphere. The medium was changed every 2–3 days for the maintenance of cells.

### 2.3. Bioactivity Assays

#### 2.3.1. Cell Viability Assay

The CellTiter-Glo^®^ Luminescent Cell Viability Assay (Promega, Madison, WI, USA) was performed to evaluate any cytotoxic effect of dairy samples on Caco-2 and HepG2 cell lines. For each cell line, 96-well plates were seeded at a density of 15,000 cells/well in the case of HepG2 and 30,000 cells/well in the case of Caco-2. After two days of growth of HepG2 and after differentiation of Caco-2, cells were treated with dairy samples (LF, whey, WBM, and non-WBM) at different concentrations (10, 5, 2, 1, and 0.5 mg/mL) or 5 µM calicheamicin as a positive control of cytotoxicity for 24 h (100 µL/well). The CellTiter-Glo^®^ Luminescent Cell Viability Assay was performed according to the manufacturer’s instructions. Luminescence was measured using the Infinite^®^ 200 PRO instrument (Tecan, Männedorf, Switzerland). All samples were analyzed in duplicate in three independent viability assays.

#### 2.3.2. Oxidative Stress Assay

The protective effect of dairy samples against oxidative stress was analyzed. In this case, 50 µM menadione was used as an oxidizing agent. To measure oxidative stress in living cells, CellRox^®^ Green Reagent (Thermo Fisher Scientific) was used, which is a fluorogenic probe. The dye penetrates cells and is not fluorescent in its reduced state but exhibits green photostable fluorescence after oxidation by ROS.

The cells were seeded at a density of 300,000 cells/mL on coverslips inserted in 24-well plates. After two days of growth for HepG2 and after differentiation for Caco-2, cells were incubated for 1 h with the four dairy samples at different concentrations (5, 1, and 0.5 mg/mL) or with 1 mM N-acetylcysteine (NAC) as a control of antioxidant activity. After the incubation time, 50 µM menadione was added for 1 h and then CellRox^®^ Green Reagent was added for 30 min. Cells were then washed and fixed with 4% paraformaldehyde (PFA). After three washes in PBS, including 30 nM DAPI to stain the cell nuclei in the last wash, coverslips were mounted and observed under a Nikon 50i fluorescence microscope (NIKON, Tokyo, Japan) equipped with a Luca S camera (Figure S1A–C). The green reagent signal intensity of each nucleus was quantified with an ImageJ macro of the image analysis software ImageJ 1.52p (National Institutes of Health, Bethesda, MD, USA). The green reagent signal intensity of the whole cell population was averaged for each condition. Results were normalized to 1 for the untreated condition. For each experiment, 150–200 cells were counted, and three independent experiments were performed.

#### 2.3.3. Genotoxicity Assay

Genotoxicity was assessed by measuring the level of gamma-H2AX (the phosphorylated form of H2AX at serine 139 that is induced after activation of the DNA damage response, noted γ-H2AX) as described previously [32]. Briefly, the same process as above was carried out, using NAC as an antigenotoxic control (Figure S1D–F). After fixation with PFA, cells were incubated for 1 h at room temperature with the γ-H2AX antibody (05–636, Sigma-Aldrich) in a dilution of 1/1000 (*v*/*v*). Subsequently, the cells were washed three times with PBS and incubated with the secondary antibodies (Alexa Fluor 546 goat anti-mouse, Thermo Fisher Scientific) diluted 1/800 (*v*/*v*) for 40 min at room temperature. Cells were washed three times with PBS, and 30 nM DAPI dye was added to the last wash to stain nuclei. Coverslips were mounted onto slides with PBS-glycerol (90%) containing 1 mg/mL paraphenylenediamine and observed under a Nikon 50i fluorescence microscope equipped with a Luca S camera, analyzing the intensity of fluorescence through ImageJ macro as described above. γ-H2AX signal intensity of each nucleus was automatically determined by an ImageJ macro. γ-H2AX signal intensity was averaged for each condition, and these results were normalized to 1 for the untreated condition. For each experiment, 150–200 cells were counted, and three independent experiments were performed.

#### 2.3.4. Bioavailability Assay

To assess bioavailability, a system of coculture of Caco-2 and HepG2 cell lines was carried out using a 24 well plate with standard transwell inserts (Corning Incorporated, New York, NY, USA) in order to mimic the intestinal-liver axis. The Caco-2 cells were seeded and grown on transwell inserts (3000 cells/transwell). Once differentiated (Figure S2), Caco-2 cells were adapted to MEM HepG2 medium, and HepG2 cells were seeded on coverslips (180,000 cells/well), associating the two cell lines (Figure S3).

After two days of co-culture, Caco-2 cells were exposed to dairy samples (LF, whey, WBM, and non-WBM) at final concentrations of 5 and 1 mg/mL and NAC (final concentration of 1 mM) and incubated at 37 °C for 24 h. Subsequently, 50 µM menadione was added for 1 h to both Caco-2 and HepG2 cells. The CellRox Green Reagent was then added to both cell lines at a final concentration of 5 µM for 30 min. Afterwards, the medium was collected from the upper and lower chambers and stored at −80 °C for further analysis. Immunofluorescence was performed as described previously [33], with minor changes. Briefly, cells were washed and fixed with 4% PFA, permeabilized with 0.5% Triton X-100, and blocked with 0.1% PBS-NP40 with 3% bovine serum albumin (BSA) for 45 min. Cells were then incubated with γ-H2AX primary antibody at a 1/1000 dilution to measure genotoxicity in the two cell lines, as detailed in the previous section. For HepG2 cells grown on coverslips, the primary antibody was incubated for 1 h at room temperature. For Caco-2 cells grown in the transwell, the primary antibody was incubated overnight at 4 °C. The secondary antibody, labeled with Alexa 546, was incubated at room temperature at a 1/800 dilution for 40 min for HepG2 cells and for 1 h for Caco-2 cells. Subsequently, cells were stained with DAPI and rinsed, then coverslips with HepG2 cells or membranes cut out from the transwell inserts with Caco-2 cells were mounted and observed under the fluorescence microscope to quantify the oxidative stress and genotoxicity, as described above. For each experiment, 150–200 cells were counted, and three independent experiments were performed.

#### 2.3.5. Proteomic Analysis

To assess the possible passage of LF from the upper to the lower compartment of the transwell through the Caco-2 cell barrier, proteomic analyses were performed (Proteomics Platform of Servicios Científico Técnicos of CIBA, IACS-Universidad de Zaragoza). For controlled quantification of proteins, the Selected Reaction Monitoring (SRM) technique was used. The digestion of samples was carried out in solution, starting with a sample of approximately 40 µg of protein. For this, 10 µL of denaturing buffer (6 M urea, 100 mM TRIS buffer, pH 7.8) was added. Cysteines were then reduced by adding 1.5 µL of 200 mM dithiothreitol for 30 min at 37 °C and alkylated with 6 µL of 200 mM iodoacetamide for 30 min in darkness. Afterwards, the samples were diluted with 50 mM ammonium bicarbonate to reach a final concentration of less than 1 M urea. Digestion was carried out overnight with trypsin (Gold Trypsin, Promega) at 37 °C in a 1:20 ratio (enzyme/protein). The reaction was stopped the next day by adding concentrated formic acid (Sigma-Aldrich).

The development of the SRM method for the quantification of the proteins of interest was carried out using Skyline software (MacCoss Lab Software, Seattle, WA, USA, version 20.2.0.343). SRM analyses were performed in a hybrid triple quadrupole-linear ion trap mass spectrometer (6500QTRAP+, Sciex, Foster City, CA, USA) coupled to a nano/micro-HPLC (Eksigent LC425, Sciex).

### 2.4. Statistical Analysis

The results are presented as the mean ± standard deviation. The statistical analysis of the results was performed using the statistical software GraphPad Prism v8.0.2 (GraphPad Software, San Diego, CA, USA). The normality of the data was checked with the Shapiro-Wilk test. To compare the means of three or more unpaired groups, an analysis of variance (ANOVA) was performed, and Dunnet’s test was used as a multiple comparison test. Data that did not follow a normal distribution were submitted to the non-parametric Kruskal-Wallis test followed by Dunn’s test as a multiple comparison test. Differences with a *p*-value ≤ 0.05 were considered statistically significant.

## 3. Results and Discussion

### 3.1. Effect of Dairy Fractions on Viability of Caco-2 and HepG2 Cells

First, we carried out viability assays on Caco-2 and HepG2 cell lines in order to identify the concentrations that may have a cytotoxic effect. Calicheamicin, a cytotoxic agent that causes DNA double-strand breaks, was used as a positive control at 5 µM.

**Figure 1:**
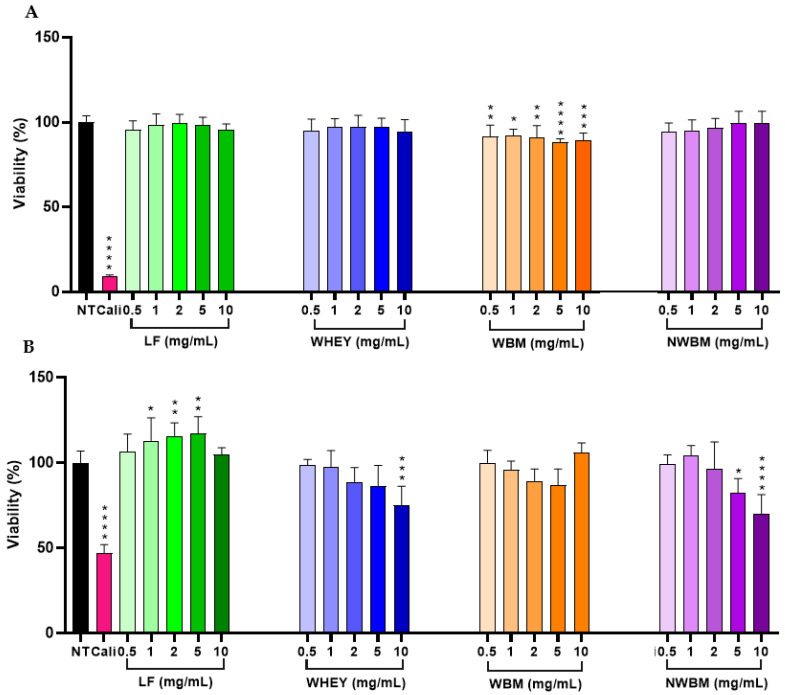
Viability of (**A**) Caco-2 and (**B**) HepG2 cells after 24 h of incubation with dairy fractions. NT: non-treated cells. Cali: positive control of cytotoxicity consisting of cells treated with 5 µM calicheamicin. LF: lactoferrin. WBM: washed buttermilk. NWBM: non-washed buttermilk. The viability is expressed in percentages with respect to NT. The values represent the mean ± standard deviation of two replicates in three independent experiments (n = 6). * *p* < 0.05, ** *p*< 0.01, *** *p* < 0.001, **** *p* < 0.0001, compared with NT.

The results obtained showed that LF, whey, and non-WBM did not affect the Caco-2 cells viability when incubated for a maximum of 24 h. However, WBM induced some cell toxicity, although without going below 88% viability (Figure 1A).

In the case of HepG2 cells, a dose-dependent response was observed for whey and non-WBM, with a significant decrease (about 30%) in cell viability for the highest concentration (10 mg/mL) when compared to non-treated cells. Surprisingly, no significant decrease was observed for WBM, in contrast to Caco-2 cells. In addition, there was also no decrease in cell viability with LF at concentrations from 1 to 5 mg/mL (Figure 1B). Some authors reported that LF may promote the proliferation of some cells, such as bone-forming cells, osteoblasts, and cartilage cells [34]. It was thought that LF supported cell proliferation due to its ability to transport iron into cells. Moreover, LF has been shown to act as a growth factor activator. Actually, the effect of LF on intestinal epithelial cells has been reported to be more potent than that of epidermal growth factor [35].

Due to a decrease in viability observed with some samples, the highest concentration of 10 mg/mL was suppressed for the rest of the tests.

### 3.2. Effect of Dairy Fractions on Oxidative Stress Caused by Menadione on Caco-2 and HepG2 Cells

Oxidative stress is the result of an imbalance between ROS production and the cells’ ability to eliminate these reactive species. Organisms have endogenous antioxidant defense mechanisms to deal with oxidative stress [7]. It may also be important to provide an exogenous source of antioxidants to complement the endogenous activity. Thus, it was shown that a high dairy diet in mice resulted in a decrease in ROS and malondialdehyde [36].

**Figure 2:**
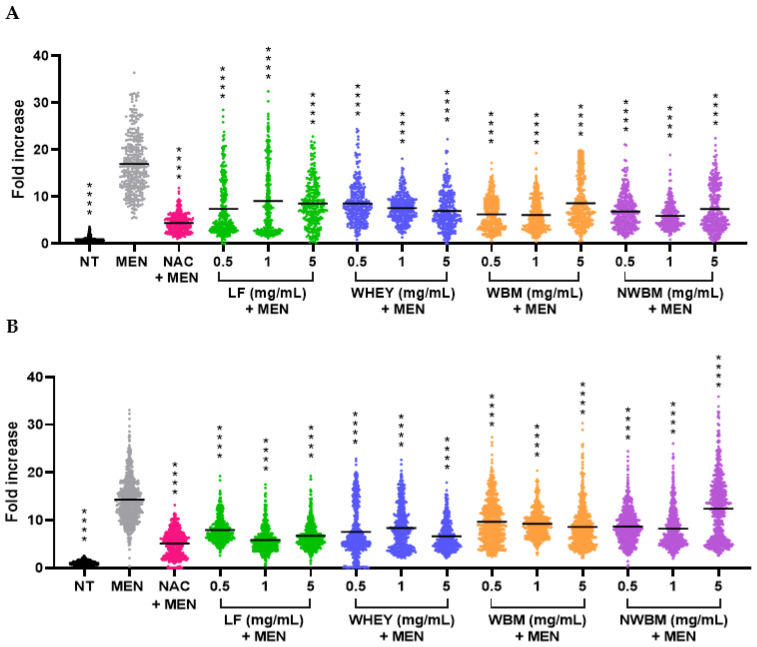
Effect of dairy fractions on oxidative stress caused by menadione on (**A**) Caco-2 and (**B**) HepG2 cells. NT: non-treated cells. MEN: positive control of oxidative stress, cells treated with 50 µM menadione for 1 h. NAC + MEN: cells treated for 1 h with 1 mM NAC as a control of antioxidant effect before menadione treatment. LF: lactoferrin. WBM: washed buttermilk. NWBM: non-washed buttermilk. The results were normalized to 1 for the untreated condition. The values represent the intensity of 150–200 cells counted in each experiment, on three independent experiments. The bars represent the means of all counts. **** *p* < 0.0001, compared with MEN.

To evaluate the effect of the different dairy fractions on oxidative stress, cells were treated with menadione as an oxidizing agent. In this assay, the fluorescent signal given by the probe that detected oxidative stress was measured. After 1 h of treatment with menadione, the fluorescent signal was highly increased for both Caco-2 cells (Figure 2A) and HepG2 cells (Figure 2B). In contrast, pre-incubation with 1 mM NAC before the menadione treatment impeded the oxidative stress response, inducing a strong decrease in the level of oxidative stress (Figure 2). Interestingly, all the dairy products tested significantly reversed the oxidative stress caused by menadione on Caco-2 (Figure 2A) and HepG2 (Figure 2B) cells.

In Caco-2 cells, whey showed a dose-dependent protective effect (Figure 2A); furthermore, dairy fractions at a concentration of 1 mg/mL (whey, WBM, and non-WBM) showed a stronger antioxidant effect compared to LF, suggesting the presence of an antioxidant compound in these fractions. In HepG2 cells, this relationship with the amount of by-product added to the cells was not observed (Figure 2B), for which the whey concentration of 0.5 mg/mL decreased oxidative stress more than the 1 mg/mL concentration. However, it is noteworthy that the highest concentration of whey (5 mg/mL) showed the greatest effect in both cell lines. It is well known that some whey components, such as α-lactalbumin, have antioxidant activity, producing a reduction in oxidative stress due to their iron chelating-properties. In addition, whey contains sulphur-rich amino acids, which may also improve antioxidant defense [7].

The highest concentration of non-WBM (5 mg/mL) did not reverse oxidative stress as properly as lower doses of non-WBM (0.5 and 1 mg/mL) in HepG2 cells (Figure 2B), when compared to Caco-2 cells (Figure 2A). This could be the result of cell cytotoxicity induced in HepG2 cells at this concentration, as shown previously (Figure 1B). In the case of the WBM fraction, all the concentrations tested had a protective effect on Caco-2 and HepG2 cells from the stress caused by menadione.

Overall, the dairy by-product with the highest antioxidant activity on Caco-2 cells was the non-WBM at a concentration of 1 mg/mL, decreasing the effect of menadione by 11-fold compared to the menadione control. This result suggests strong antioxidant activity in this dairy fraction. A similar effect was shown by WBM at the same concentration, with a 10.8-fold decrease in respect to menadione (Figure 2A). However, in HepG2 cells, the greatest antioxidant effect was shown with 1 mg/mL of LF (Figure 2B), which reduced menadione stress by 8.4-fold, almost as much as NAC (9-fold decrease over menadione control). These results were satisfying since the accepted dose for the addition of LF to food is around 1 mg/mL [16]. On HepG2 cells, whey and non-WBM at 1 mg/mL reduced menadione stress by 5.9 and 6-fold, respectively.

In the study by Buey et al. [26], the effect of these dairy by-products on oxidative stress caused by the lipopolysaccharide (LPS) present in the membranes of gram-negative bacteria was analyzed. They showed that both whey and LF had antioxidant power, reducing lipid oxidation and protein damage caused by LPS. In the same study, BM did not seem to reduce the oxidative stress caused by LPS, while in our study, this dairy by-product did protect cells against the oxidative effect of menadione. Furthermore, Prakash et al. [37] affirmed that protein hydrolysates from whey and BM contain antioxidant agents and enhancers of phagocytic activity in macrophages. As in our experimental set-up, the dairy fractions were used without hydrolysis, so proteins may not enter the cells. However, proteins may pass through Caco-2 cells through the paracellular route or via transcytosis [38]. In addition, some specific transport mechanisms may be involved, such as intelectin 1, a LF-receptor, expressed in Caco-2 cells [39]. It has also been shown that LF binds to the asialoglycoprotein receptor (ASGPR) in rat liver [40]. Yet, small molecules such as amino acids [27] or other small metabolites—to be identified—could explain the antioxidant effect observed here. Further studies are needed to characterize these compounds.

### 3.3. Effect of Dairy Fractions on Genotoxicity Caused by Menadione on Caco-2 and HepG2 Cells

Human cells are continuously exposed to physiological and external influences that can cause cytotoxic, oxidative, and genotoxic damage. When an imbalance occurs in the dynamics between ROS generation and antioxidant systems, oxidative damage to all cellular targets, including DNA, arises [41]. HepG2 cells are capable of performing oxidative metabolism of nutrients, metabolites, and xenobiotics. Furthermore, the intestine is exposed to high levels of ingested oxidants, and Caco-2 cells show many functions similar to those of enterocytes [42].

**Figure 3:**
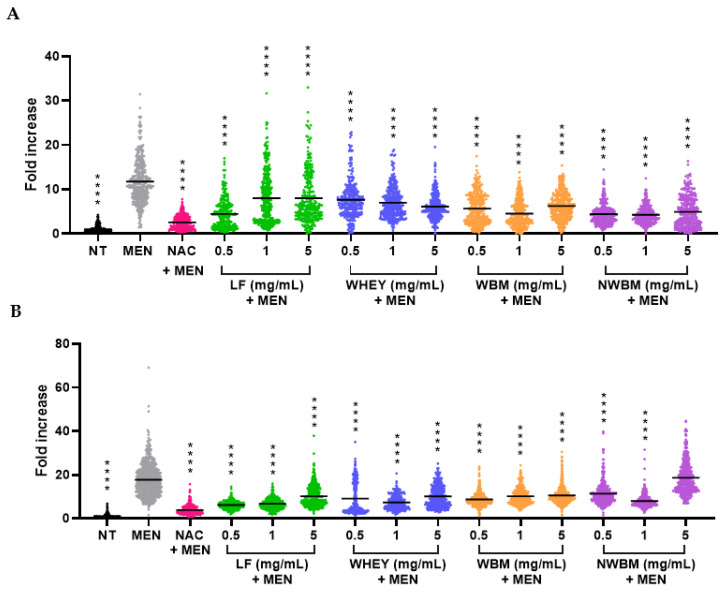
Effect of dairy fractions on genotoxicity caused by menadione on (**A**) Caco-2 and (**B**) HepG2 cells. NT: non-treated cells. MEN: positive control of genotoxicity, cells treated with 50 µM menadione for 1 h. NAC + MEN: cells treated with 1 mM NAC as a control of antigenotoxic effect before treatment with 50 µM menadione for 1 h. LF: lactoferrin. WBM: washed buttermilk. NWBM: non-washed buttermilk. The results were normalized to 1 for the untreated condition. The values represent the intensity of 150–200 cells counted in each experiment, on three independent experiments. The bars represent the means of all counts. **** *p* < 0.0001, compared with MEN.

Therefore, we have analyzed the protective effect of dairy by-products against DNA damage indirectly caused by menadione after the generation of reactive species that finally generate DNA damage. To evaluate the effect of the different dairy fractions on a genotoxic stress, we again used menadione treatment as a positive control. Under these conditions, after immunostaining, we quantified the fluorescent signal due to γ-H2AX, a classic biomarker of DNA damage. We observed a strong induction of DNA damage after the menadione treatment (Figure 3). NAC used as an antioxidant allowed the reversion of the γ-H2AX signal, indicating that DNA damage was caused indirectly through oxidative stress (Figure 3). Overall, the dairy products significantly reversed the genotoxic stress caused by menadione on Caco-2 (Figure 3A), with whey showing the same dose-dependent effect as observed in the antioxidant capacity analysis (Figure 2A).

The dairy sample with the highest antigenotoxic activity in Caco-2 cells was non-WBM at low concentrations, followed by LF at 0.5 mg/mL (below the maximum allowable dose as detailed in Regulation (EC) No 258/97) [16] and WBM at 1 mg/mL (Figure 3A), reducing the genotoxic effect of menadione by about 7.3-fold. For HepG2 cells, the greatest DNA protector was LF at 0.5 mg/mL (Figure 3B). with an effect inversely proportional to the concentration. The same dose-response relationship was shown by WBM. Altogether, our results suggest that some antioxidant compounds present in the tested dairy fractions may help prevent DNA lesions associated with oxidative stress.

Several studies on the antigenotoxic effect of fermented milk against mutagens have been condensed in an extensive review [43]. In this review, in vivo studies with rodents fed fermented milk showed a reduction in the incidence of carcinogen-induced colon cancer tumors and a decrease in DNA damage. In addition, the in vitro studies reviewed also demonstrated the antigenotoxic and antimutagenic effects of fermented milk [43].

The results of Chiang et al. [44] showed that hydrolyzed bovine colostrum whey presented oxidative damage inhibitory activities and an inhibitory effect on the decomposition of supercoiled DNA. In addition, in the study by Tian [45], the antigenotoxic properties of LF were determined in HT29 cells, a model of human intestinal cells, after DNA had been damaged by fecal water, finding the greatest effect of LF at high concentrations of 10 and 20 mg/mL. Habib et al. [35] also studied the antioxidant and DNA damage inhibitory activities of LF. They used camel milk LF and obtained results similar to those reported here. They analyzed the complete degradation of plasmid DNA treated with UV light, H_2_O_2_ and FeSO_4_ and how DNA damage was reduced in the presence of LF, most likely through binding catalytic iron. Furthermore, Ogasawara et al. [46] demonstrated that native LF and iron saturated LF clearly protected DNA from fragmentation caused by UV radiation in the presence of ROS. All these results indicated that LF could suppress ROS and protect DNA from damage.

In conclusion, we show here (Figure 2 and Figure 3) that dairy by-products partially rescue the menadione effects (oxidative stress and subsequent genotoxic stress). The remaining oxidative stress could explain the remaining genotoxicity. It is possible that a treatment with a lower concentration of menadione would have allowed total protection against both oxidative and genotoxic stresses.

### 3.4. Bioavailability of Dairy Fractions in Caco-2/HepG2 Coculture

**Figure 4:**
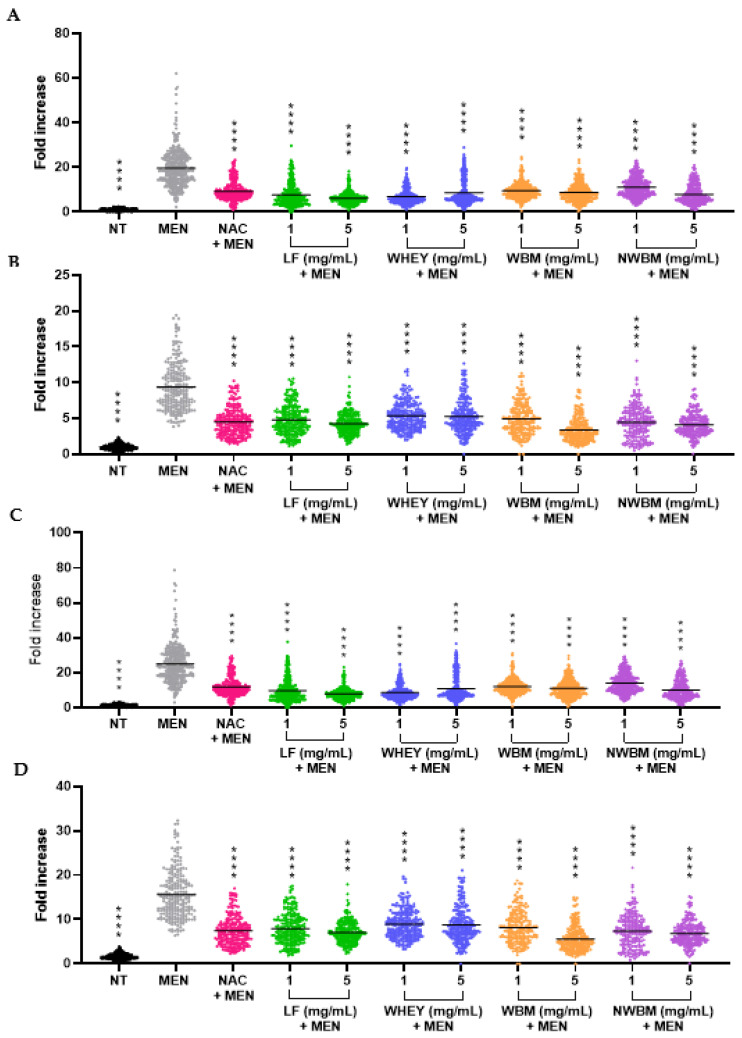
Effect of dairy fractions on oxidative stress (**A**,**B**) and genotoxicity (**C**,**D**) caused by menadione on Caco-2 cells (**A**,**C**) and HepG2 cells (**B**,**D**) co-cultured in standard transwell inserts. NT: non-treated cells. MEN: positive control of oxidative stress and genotoxicity, cells treated with 50 µM menadione. NAC + MEN: cells treated with 1 mM NAC as a control of antioxidant and antigenotoxic effects before treatment with 50 µM menadione. LF: lactoferrin. WBM: washed buttermilk. NWBM: non-washed buttermilk. The results were normalized to 1 for the untreated condition. The values represent the intensity of 150–200 cells counted in each experiment, on three independent experiments. The bars represent the means of all counts. **** *p* < 0.0001, compared with MEN.

In order to study the bioavailability and metabolism of the different dairy compounds, a Caco-2/HepG2 co-culture intestinal cell monolayer model was used. This co-culture was chosen to model the intestinal-liver axis and study the transport and changes in the functional activity of the metabolites. This co-culture system has already been used previously [47]. To understand the behavior of the dairy by-products across a barrier similar to the intestinal epithelium, we evaluated the transport and metabolism of dairy samples in differentiated Caco-2 cells and their exposure to HepG2 cells, in order to analyze their bioavailability and their effects on oxidative stress and DNA damage caused by menadione (Figure 4). In addition, the proteomic analysis of the culture medium allowed us to confirm the passage of LF from the upper transwell to the lower compartment. The amount of LF detected was 63-fold higher in the upper chamber (in contact with the apical side of Caco-2 cells) than in the lower chamber (in contact with HepG2 cells).

Analyzing the bioavailability and effect of the samples, we noticed that menadione-induced oxidative stress was reverted by the different dairy fractions in Caco-2 cells (Figure 4A) but also in HepG2 cells (Figure 4B). This meant that at least part of the antioxidant compounds in the dairy products were bioavailable and protected HepG2 cells from menadione stress. The reversion patterns of the genotoxic stress in both cell lines led us to the same conclusion (Figure 4C,D). This confirms that menadione effects (oxidative stress and genotoxicity) are closely related, with cells displaying the highest level of oxidative stress being those displaying an increased DNA damage level. It is also observed that the protective effect of milk samples was effective against both stresses, strongly suggesting that the antioxidant effect impeded both oxidative and genotoxic stresses. This study also shows that these potential antioxidant compounds contained in the dairy products were not altered and maintained their activity when they crossed the Caco-2 barrier and reached HepG2 cells on the basal side, similar to that observed in the single cell model (see Figure 2 for oxidative stress and Figure 3 for genotoxic stress).

The single Caco-2 epithelial model lacks communication with other organs involved in the regulation of compound absorption, which may be a limitation for this model. However, the co-culture model with both Caco-2 and HepG2 cell lines could improve research on nutrient absorption [48]. Caco-2/HepG2 coculture models have been used previously to analyze the intestinal transport of polyphenol extract [47] or for in vitro iron absorption studies [48]. In the study by Scheers et al. [48], iron uptake on the apical surface of Caco-2 cells was higher in the co-culture model with HepG2 than in the Caco-2 cell monoculture. For the design of a successful combined liver and intestine model, some requirements must be considered. The chosen liver cell line should be easily accessible, have similar nutritional requirements to those of Caco-2 cells, and have reproducible secretion; these are prerequisites ensuring that the cells will not lose their phenotypes in culture, as occurs with primary liver cell lines [48]. For all these reasons, the chosen HepG2 cell line is a good choice for co-culture with Caco-2 cells.

In the present study, we have analyzed the antioxidant and antigenotoxic activity of dairy fractions and isolated proteins, although we have not considered what would occur in the case of a dynamic process within the gastrointestinal tract. However, we should point out that this study is the first part of a wider research, in which the effect of those samples after digestion will be investigated by analyzing their antioxidant and antigenotoxic potential in such conditions. Even though no digestion assay was performed, we observed antioxidant and antigenotoxic activities of all dairy fractions in the HepG2 cells when grown in co-culture with Caco-2 cells, suggesting that small compounds may play this role.

## 4. Conclusions

In conclusion, lactoferrin, whey, and buttermilk (washed and non-washed) display antioxidant and protective effects against DNA damage in Caco-2 and HepG2 cell lines. The co-culture between these two cell lines has made it possible to show the transfer of compounds that exert these protective functions on both cell lines.

Therefore, this study provides useful information to increase the commercial value of dairy by-products as multifunctional ingredients and their recognition as a natural source of antioxidants. It would be interesting to continue investigating the protective activity of milk compounds against genotoxicity as well as their bioavailability, since there are not many studies on this subject. Further characterization of the active compounds contained within these fractions will be key in the future.

## Data Availability

The data presented in this study are available on request from the corresponding author. The data are not publicly available due to reasons of privacy.

## Supplementary Materials

The following supporting information can be downloaded at: https://www.mdpi.com/article/10.3390/foods12102073/s1. Figure S1: (A, B, C) Green fluorescence emitted by CellRox^®^ Green Reagent (10×) (Bars, 1 µm) and (D, E, F) γ-H2AX signal (40×) (Bars, 10 µm) in (A, D) untreated Caco-2 cells, (B, E) Caco-2 cells treated with 50 µM menadione and (C, F) Caco-2 cells incubated with 1 mM NAC before treatment with 50 µM menadione. In HepG2, the same signal intensity was detected for each case; Figure S2: (A) Scheme of differentiated Caco-2 cells showing cellular structures (tight junction, nucleus) and confocal plans for the z-stack analysis. Cells were grown on transwell membranes (dotted line) for 21 days. (B, C, D) Confocal microscopic images (40×) of fixed and stained differentiated Caco-2. Cells were stained (B) with ZO-1 (green) for tight junction analysis and (C) with DAPI (blue) for nuclei staining. (D) Confocal analysis for the middle cellular plan shows an intermediate staining. (E) Close up of the confocal middle plan. Bars, 10 μm; Figure S3: Bicameral chamber used for co-culture of cell lines, mimicking the intestinal-liver axis, with Caco-2 cells seeded on the standard transwell insert and HepG2 cells seeded on the coverslip at the bottom. Graphics elaborated with BioRender.com.
