# Supplementary material for: Dairy By-Products and Lactoferrin Exert Antioxidant and Antigenotoxic Activity on Intestinal and Hepatic Cells
Source: Foods. 2023 May 21;12(10):2073. doi: 10.3390/foods12102073 (PMC10217426; doi:10.3390/foods12102073)
Supplement: Supplementary file 1 [file foods-12-02073-s001.zip › foods-2382946-supplementary.pdf]

APPENDIX A. Supplementary material

A.1. Oxidative stress and genotoxicity assays

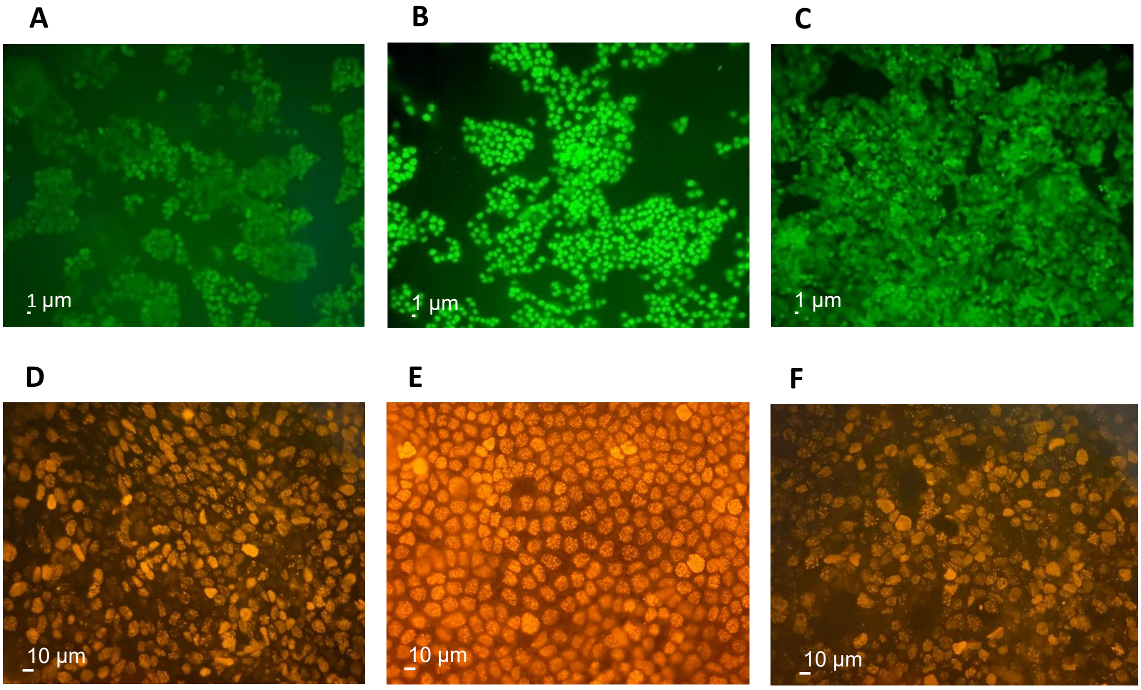

Figure S1. (A, B, C) Green fluorescence emitted by CellRox® Green Reagent (10x) (Bars, 1 μm) and (D, E, F) γ-H2AX signal (40x) (Bars, 10 μm) in (A, D) nontreated cells, (B, E) cells treated with 50 μM menadione and (C, F) cells incubated with 1 mM NAC before treatment with 50 μM menadione.

A.2. Differentiation of Caco-2 cells

Differenciated Caco-2 cells were grown as monolayers for 17 days on transwell membranes. To ensure differentiation and the monolayer formation, cells were washed with PBS, fixed with 4% formaldehyde in PBS for 20 min, blocked and permeabilized in PBS containing 3% BSA and 0.3% Triton X-100 at room temperature for 30 min. Subsequently, monolayers were incubated with PBS containing 0.1% Tween-20, 2% BSA, rabbit anti-zonula occludens 1 (ZO-1) (1:25, v/v) antibodies (rabbit polyclonal antibody (40-2300), Thermo Fisher Scientific) and incubated overnight at 4°C. The incubation was followed by three washes in PBS and incubation for 1 h at room temperature in Alexa Fluor 488 labeled secondary antibody (1:500, v/v) and counterstained with DAPI. ZO-1 and DAPI signals were visualized with a Leica SP8 confocal microscope and software (Leica Microsystems, Wetzar, Germany). Two independent experiments were conducted and independent fields examined.

Figure S2 shows the thigh junctions between cells marked with anti-ZO-1, and nuclei counterstained with DAPI. Cells were arranged in a monolayer, showing a polarized structure with nuclei at the basal side, and ZO-1 at the apical side.

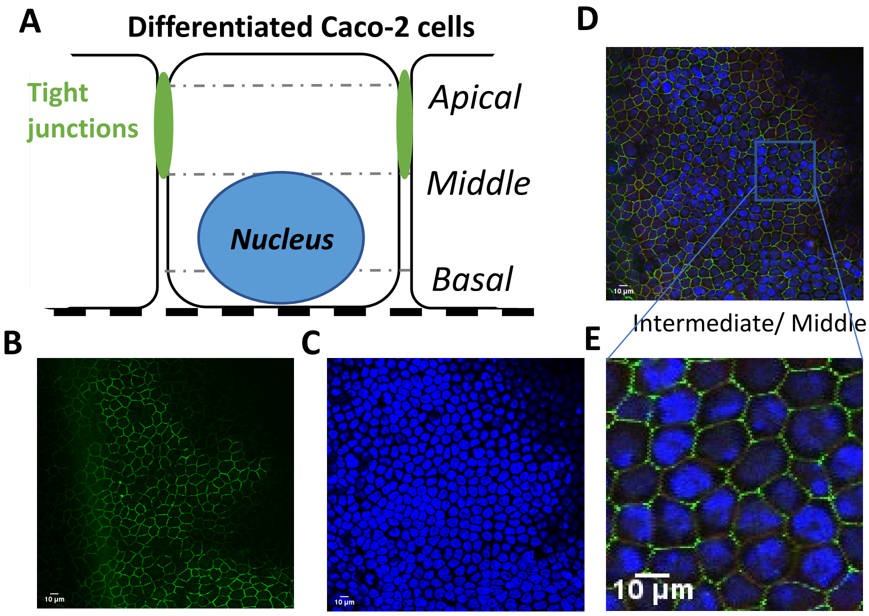

Figure S2. (A) Scheme of differentiated Caco-2 cells showing cellular structures (tight junction, nucleus) and confocal plans for the z-stack analysis. Cells were grown on transwell membranes (dotted line) for 21 days. (B, C, D) Confocal microscopic images (40x) of fixed and stained differentiated Caco-2. Cells were stained (B) with ZO-1 (green) for tight junction analysis and (C) with DAPI (blue) for nuclei staining. (D) Confocal analysis for the middle cellular plan shows an intermediate staining. (E) Close up of the confocal middle plan. Bars, 10 µm.

A.3. Bioavailability assay

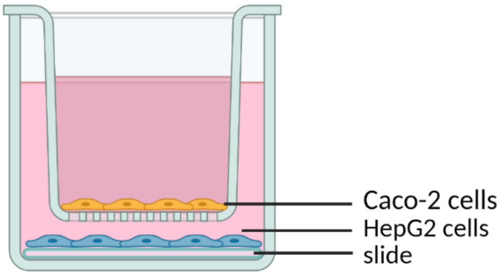

Figure S3. Bicameral chamber used for co-culture of cell lines, mimicking the intestinal-liver axis, with Caco-2 cells seeded on the standard transwell insert and HepG2 cells seeded on the coverslip at the bottom. Graphics elaborated with BioRender.com.
